# Interplay between the Chaperone System and Gut Microbiota Dysbiosis in Systemic Lupus Erythematosus Pathogenesis: Is Molecular Mimicry the Missing Link between Those Two Factors?

**DOI:** 10.3390/ijms25115608

**Published:** 2024-05-21

**Authors:** Alessandra Maria Vitale, Letizia Paladino, Celeste Caruso Bavisotto, Rosario Barone, Francesca Rappa, Everly Conway de Macario, Francesco Cappello, Alberto J. L. Macario, Antonella Marino Gammazza

**Affiliations:** 1 Department of Biomedicine, Neurosciences and Advanced Diagnostics (BiND), University of Palermo, 90127 Palermo, Italy; letizia.paladino@unipa.it (L.P.); celeste.carusobavisotto@unipa.it (C.C.B.); francesca.rappa@unipa.it (F.R.); francesco.cappello@unipa.it (F.C.); antonella.marinogammazza@unipa.it (A.M.G.); 2 Euro-Mediterranean Institute of Science and Technology (IEMEST), 90139 Palermo, Italy; econwaydemacario@som.umaryland.edu (E.C.d.M.); ajlmacario@som.umaryland.edu (A.J.L.M.); 3 Department of Microbiology and Immunology, School of Medicine, University of Maryland at Baltimore-Institute of Marine and Environmental Technology (IMET), Baltimore, MD 21202, USA

**Keywords:** systemic lupus erythematosus, chaperone system, gut microbiota, leaky gut, autoimmunity, molecular mimicry, chaperonopathy, chaperonotherapy

## Abstract

Systemic lupus erythematosus (SLE) is a multifactorial autoimmune disease characterized by self-immune tolerance breakdown and the production of autoantibodies, causing the deposition of immune complexes and triggering inflammation and immune-mediated damage. SLE pathogenesis involves genetic predisposition and a combination of environmental factors. Clinical manifestations are variable, making an early diagnosis challenging. Heat shock proteins (Hsps), belonging to the chaperone system, interact with the immune system, acting as pro-inflammatory factors, autoantigens, as well as immune tolerance promoters. Increased levels of some Hsps and the production of autoantibodies against them are correlated with SLE onset and progression. The production of these autoantibodies has been attributed to molecular mimicry, occurring upon viral and bacterial infections, since they are evolutionary highly conserved. Gut microbiota dysbiosis has been associated with the occurrence and severity of SLE. Numerous findings suggest that proteins and metabolites of commensal bacteria can mimic autoantigens, inducing autoimmunity, because of molecular mimicry. Here, we propose that shared epitopes between human Hsps and those of gut commensal bacteria cause the production of anti-Hsp autoantibodies that cross-react with human molecules, contributing to SLE pathogenesis. Thus, the involvement of the chaperone system, gut microbiota dysbiosis, and molecular mimicry in SLE ought to be coordinately studied.

## 1. Introduction

The chaperone system (CS) is composed of molecular chaperones, some of which are heat shock proteins (Hsps), co-chaperones, chaperone co-factors, and chaperone interactors and receptors [1]. The canonical functions of the CS are directed to the maintenance of protein homeostasis and, for these functions, it interacts with the ubiquitin-proteasome system (UPS) and with the chaperone-mediated autophagy (CMA) machinery [2,3,4]. Chaperones perform their canonical functions not alone, as monomers, but in teams, which are oligomers made up of identical subunits, e.g., Hsp60, or constituted of non-identical but similar subunits, e.g., CCT [5,6,7]. Furthermore, the teams interact between themselves and form functional networks, e.g., Hsp70/DnaK-Hsp40/DnaJ-Prefoldin [8,9].

In the last few years, increasing evidence has pointed out the “other side of the coin” of the CS. In fact, when abnormal in structure/function/location/concentration, its members may become etiopathogenetic factors, causing diseases known as the chaperonopathies [10,11,12].

The involvement of Hsps in autoimmunity has been investigated for many years, and autoimmune diseases (ADs) can be classified into the group of chaperonopathies by mistake or collaborationism, i.e., acquired chaperonopathies in which a chaperone functions to favor the pathogenic mechanism and leads to disease [5,10,11,12,13,14,15]. However, their role is still under investigation. They may either promote immune cell activation and pro-inflammatory cytokine production and act as autoantigens eliciting autoantibodies, or perform a pro-immune tolerance restoring activity [16,17]. Depending on the role, Hsps have been proposed for the development of novel therapeutic strategies (positive or negative chaperonotherapy) [18,19,20]. Among the ADs in which the CS members, especially the Hsps, are believed to play a role is systemic lupus erythematosus (SLE) [21]. SLE is a chronic, multisystemic autoimmune/inflammatory disease affecting almost every organ and tissue of the body, with multiple clinical manifestations ranging from milder symptoms, such as skin rashes or non-erosive arthritis, to more serious and potentially life-threatening complications mostly affecting the kidney and the central nervous system [22,23,24,25]. SLE can affect persons of all ages and ethnic groups and both sexes. However, more than 90% of newly diagnosed cases are women in their childbearing years, with a female-to-male ratio of 9–10:1 [26]. On the contrary, men are diagnosed at a more advanced age and often show a more severe phenotype, with an overall higher risk for progression into SLE complications such as lupus nephritis (LN) and end-stage renal disease (ESRD) [26,27].

A distinctive hallmark of SLE is the breakdown of self-tolerance and the production of various autoantibodies, including antinuclear antibodies (ANAs) [28]. The interaction between autoantibodies and self-antigens produces immune complexes, which occur in circulation or localize in multiple tissues, triggering inflammation and complement activation causing immune-mediated organ damage [29]. SLE etiopathogenesis and molecular mechanisms remain largely unknown. However, numerous findings suggest a genetic predisposition to SLE development acting together with a combination of immunological, endocrine, and environmental factors [30,31,32,33].

In the last few years, the role of the gut microbiota has been investigated in the etiopathogenesis of SLE and other ADs. A normal/healthy gut microbiota contributes to the development of a functioning immune system [34]. On the contrary, gut microbiota alteration (dysbiosis) can result in the breakdown of immune tolerance, the over-activation of T cells, and the production of pro-inflammatory cytokines. All these events, in turn, can activate autoimmune responses, leading to the development of ADs [34]. Numerous findings suggest that the reason for this relationship may reside in the bacterial metabolites/products translocation from the intestinal lumen into the circulation because of increased intestinal permeability [35].

Here, we provide an overview of the involvement of the CS and gut microbiota dysbiosis in SLE pathogenesis, suggesting molecular mimicry as a potential link between them. A detailed understanding of the relationship between these three factors will likely contribute to the identification of novel promising biomarkers and therapeutic targets.

## 2. The Chaperone System and SLE

The role of the CS in SLE etiopathogenesis is multifaceted and not yet fully understood. Three conditions have elicited particular interest: (i) Hsps’ overexpression; (ii) the production of anti-Hsp autoantibodies, and (iii) Hsps’ presence on the surface of peripheral blood mononuclear cells (PBMCs), which correlates with high disease activity [36,37]. 

Higher levels of Hsp90 were found in PBMCs of patients with active SLE compared to patients with inactive disease, age- and sex-matched healthy controls, or patients who suffered from rheumatoid arthritis [38,39]. Similarly, Hsp70 levels were found to be elevated in PBMCs from SLE patients compared with those from healthy age- and sex-matched volunteers [38]. However, there was no correlation between the two Hsps, and only Hsp90 levels positively correlated with disease activity and onset [40]. The early increased levels of Hsp90 in some SLE patients is primarily dependent upon the enhanced transcription of the *HSP90β* gene, suggesting the activation of a specific gene program underlying the pathogenic mechanism of the disease [40,41,42]. On the contrary, the later elevation of Hsp70 levels is attributed to a stress response against the ongoing disease process [40,42]. Similarly to Hsp70, Hsp27 levels are also associated with disease activity. Both Hsp70 and Hsp27 levels were investigated in the renal tissue of patients with different forms of LN (diffuse proliferative, focal proliferative, and membranous) and were found within the cytoplasm of tubular epithelial cells of all patients [40]. A significant positive correlation was found between Hsp27 levels and disease severity in patients with diffuse proliferative nephritis [43].

PBMCs (lymphocytes and monocytes) from SLE patients not only had elevated intracellular levels of Hsp90, but also elevated levels on their surface, suggesting its role as an autoantigen leading to the production of autoantibodies [44,45]. Autoantibodies (IgM and IgG) against Hsp90 were found in the sera from a significant proportion of patients with SLE, both adults and children, compared to healthy controls [46,47,48]. Adults carrying higher antibody levels were more likely to have renal disease following an intense deposition of the protein in subepithelial, subendothelial, and mesangial areas of the glomeruli [47,49]. 

Higher levels of Hsp90 and anti-Hsp90 autoantibodies in the sera from SLE patients were also associated with higher levels of IL6 [50]. Both IL6 and IL10 have been found to be higher in SLE patients and positively correlated with disease activity and complications [51,52,53]. Both cytokines induce the transcription of the *HSP90β* gene in cultured PBMCs [54,55]. Elevation of these cytokines in SLE patients may induce an increase in Hsp90 levels, both intracellularly and on the surface of cells, which, in turn, leads to autoantibody production [55,56]. These results suggest that Hsp90 contributes to disease onset and progression, and to the establishment of inflammation. Therefore, targeting Hsp90 to diminish its levels may be a promising therapeutic treatment to delay disease progression [57]. For instance, in an SLE mouse model, it was observed that chemical treatment targeting the surface translocation of gp96 diminished and alleviated SLE-associated manifestations, like glomerulonephritis, proteinuria, and levels of antinuclear and DNA antibodies. All of this was accompanied by a reduction in the maturation of dendritic cells (DCs) and antigen-presenting cells, and by the activation of B and T cells [58]. The administration of a DNA vaccine encoding Hsp90 induced tolerogenic immune responses, with a reduction in anti-dsDNA autoantibody production, that limited SLE manifestations (e.g., renal disease) and extended the survival in lupus-prone mice [59]. Similar outcomes were obtained using a DNA vaccine encoding Hsp70 [60].

All these results suggest that chaperonotherapy may be effective, namely, a treatment strategy consisting of inhibiting/eliminating (negative chaperonotherapy) or boosting/replacing (positive chaperonotherapy) the pathogenic chaperone. For instance, it has been reported that the small heat shock protein (sHsp), alpha-B crystallin (HSPB5; CRYAB), attenuates the severity and disease progression of LN in lupus-prone mice (positive chaperonopathy) [18,61].

## 3. The Gut Microbiota in SLE 

The gut microbiota is a complex population composed of a large number of commensal microorganisms (some estimates reach 100 trillion) residing in the gastrointestinal tract, which has co-evolved with its host and provides benefits to it in multiple ways, including digestion, the production of nutrients, and detoxification, ensuring a complex and mutual beneficial relationship [62]. The gut microbiota plays a key role in the biology and homeostasis of cells of the innate and adaptive immune system. Therefore, an imbalance in the quantity and/or quality of its composition, including a loss of beneficial bacteria, an excessive growth of potentially harmful bacteria, or a loss of overall bacterial diversity, i.e., dysbiosis, may trigger autoimmunity [34,63,64,65]. Dysbiosis has been primarily associated with inflammatory bowel diseases (IBDs), such as Crohn’s disease (CD) and ulcerative colitis (UC) [66]. Several studies have demonstrated the association between an imbalance of the gut microbiota and the etiopathogenesis of extra-intestinal diseases, including autoimmune diseases such as SLE [67,68,69,70].

The dynamics of the gut microbiota has been investigated in a murine lupus-prone model, and differences in the composition and overall diversity were found compared to healthy controls [71]. The gut microbiota was different in males as compared to females, with an over-representation of *Lachnospiraceae* in females that was associated with an earlier onset and more severe manifestation of SLE [71,72]. This was taken as evidence that sex affects the disease course, likely because of the control exerted by sex hormones in the regulation of the immune system. The use of probiotic lactobacilli and retinoic acid as dietary supplements improved symptoms, suggesting that this type of treatment could be efficacious in relieving inflammatory flares in lupus patients [71].

In a mouse LN model, the lack of *Lactobacillus* occurred before (not after) disease onset, suggesting its involvement in disease pathogenesis, and conversely, restoration of the *Lactobacillus* population enhanced the gut mucosal barrier, suppressed gut inflammation, and attenuated LN, prolonging mice survival [73]. However, *Lactobacillus* played an opposite role in studies performed with different lupus mouse models. For instance, the gut microbiota changed before and after disease onset in lupus-prone mice, with an increase in specific genera during disease progression [74]. A positive correlation between the abundance of *Lactobacillus* species and poorer renal function and higher-level systemic autoimmunity was observed. 

**Figure 1:**
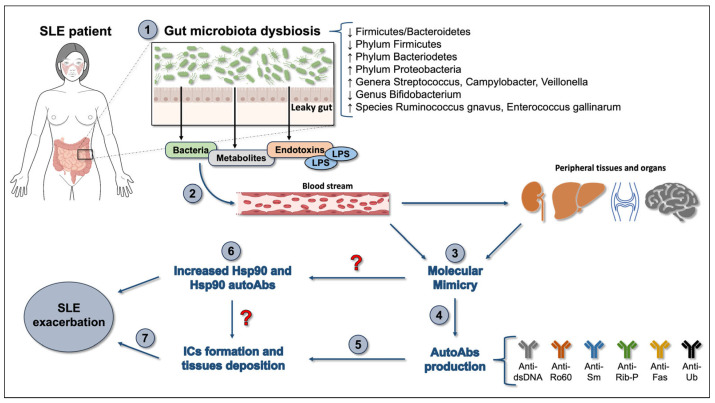
An overview of the role of the gut microbiota in SLE. Gut microbiota dysbiosis in SLE patients shows a significant reduction in both the richness and diversity of the gut microbiota, including a lower Firmicutes/Bacteroidetes ratio (1). Gut microbiota dysbiosis may cause an increase in intestinal permeability (leaky gut) and favor the translocation of pathogens and their products/metabolites from the intestinal lumen to the systemic circulation and thereby to other organs (2), resulting in inflammation and antigenic cross-reactivity via the mechanism of molecular mimicry (3). The translocation of the gut commensal autoantigen-mimicking peptides induces the production of autoantibodies, such as anti-Ro60, anti-Sm, anti-dsDNA, anti-Rib-P, anti-Fas, and anti-Ub, as shown (4). These antibodies cross-react with self-antigens, forming immune complexes that deposit in peripheral tissues (5), exacerbating SLE conditions (7). This event could explain the increase in the Hsp and anti-Hsp antibody levels observed both in the circulation and in the peripheral tissues of SLE patients (6). Abbreviations: AutoAbs, autoantibodies; Anti-dsDNA, anti-double-stranded DNA; Anti-Fas, Anti- FS-7-associated surface antigen; Anti-Rib-P, anti-ribosomal-P; Anti-Ro60, RNA-binding 60 kDa Ro; Anti-Sm, anti-Smith; AntiUb, anti-ubiquitin; LPS, lipopolysaccharide; SLE, systemic lupus erythematosus.

The association between gut microbiota dysbiosis and SLE pathogenesis has a genetic basis, since fecal microbiome transplantation from SLE mice induced significant changes in immune cell distribution and overall changes in their genetic profiles, with an upregulation of certain lupus susceptibility genes [75]. Similarly, in humans, clear differences in the composition and richness of the gut microbiota were also observed between SLE patients and healthy controls, and numerous findings have suggested that gut microbiota dysbiosis is one of the mechanisms underlying SLE pathogenesis (Figure 1) [76,77,78,79,80,81,82,83].

A human healthy gut microbiota primarily consists of the phyla Firmicutes, Bacteroidetes, Actinobacteria, Proteobacteria, Fusobacteria, and Verrucomicrobia, with Firmicutes and Bacteroidetes being the most abundant [84,85,86,87]. The Firmicutes/Bacteroidetes ratio is altered in various disorders [88,89] and is affected by the diet [90]. In SLE patients, marked dysbiosis was observed, with a significant decrease in the Firmicutes/Bacteroidetes ratio as compared with healthy controls (HCs) [76,77,78,83], and with the enrichment of the phylum Proteobacteria [74,77,80,82]. The reduced Firmicutes/Bacteroidetes ratio in SLE patients was correlated with lymphocyte activation and Th17 differentiation from naïve CD4(+) lymphocytes, favoring inflammatory mechanisms [78]. Conversely, the enrichment of the gut microbiota with bacterial strains belonging to the Firmicutes phylum reduced the IL-17/IFNγ balance and prevented the over-activation of CD4^+^ lymphocytes. This suggests that supplementation with probiotics containing Treg-inducer strains able to restore the Treg/Th17/Th1 balance would be a beneficial treatment for SLE patients [78]. An imbalance of pro-inflammatory and anti-inflammatory T cells in SLE patients was observed that was correlated to changes in the intestinal microbial population [82]. 

Differences in gut microbiota dysbiosis were observed in SLE patients with active disease compared to those with inactive disease. For instance, an abundance of the genera *Streptococcus*, *Campylobacter*, and *Veillonella* and a decrease in the genus *Bifidobacterium* were observed [79]. Other authors have reported increased *Desulfovibrio piger*, *Bacteroides thetaiotaomicron,* and *Ruminococcus gnavus* species and decreased Bacilli class and *Ruminococcaceae* and *Lactobacillaceae* families in active SLE patients compared to inactive SLE patients [83]. However, one study found no significant differences in the *Firmicutes/Bacteroidetes* ratio between SLE patients and healthy controls [74], confirming the high variability in the human gut microbiota already observed in mouse models and the impossibility to outline a universally valid profile that would distinguish SLE patients from healthy controls.

## 4. Molecular Mimicry, Hsps, and Gut Microbiota Dysbiosis in SLE

The breakdown of self-tolerance plays a critical role in the occurrence and development of SLE, leading to the production of autoantibodies and the formation of cytotoxic immune complexes triggering immune and inflammatory responses [28]. All these events are common among different autoimmune conditions and may be triggered by an infection via the molecular mimicry mechanism [91]. The term molecular mimicry describes the sharing of antigens between a parasite and its host, which facilitates the evasion of the host’s immune response and the establishment of immunological tolerance [92]. In recent years, the phenomenon was often associated with autoimmunity. Amino acid sequence or structural similarities between foreign antigens and self-antigens may favor the activation of autoreactive T or B cells, resulting in autoimmune responses in some susceptible individuals [93]. The hypothesis of post-infection pathogenic events caused by molecular mimicry has been proposed to explain SLE etiopathogenesis, and various pathogens have been identified as possible culprits [94,95,96,97,98].

The evolutionary conservation of Hsps in prokaryotes and eukaryotes suggests the involvement of a molecular mimicry mechanism in the production of anti-Hsp autoantibodies in a variety of autoreactive disorders, including SLE [99,100]. For instance, high cross-reactivity was reported between isolated SLE IgGs and Hsp70 and other intracellular proteins from *Mycobacterium tuberculosis* [101]. The sera from SLE patients contain IgGs that bind to Hsp60 present on the surface of epithelial cells, favoring phosphatidylserine exposure and cell apoptosis [102]. Also, proteins and metabolites of commensal bacteria of the gut can mimic autoantigens and induce autoimmunity through molecular mimicry [35]. The impairment of the barrier function of the intestinal epithelium, which augments intestinal permeability (leaky gut), may favor the translocation of bacteria and bacterial components, such as lipopolysaccharides (LPSs) and endotoxins, from the intestinal lumen to the systemic circulation which thereby reach other organs [103]. These bacterial components, in turn, may act as cross-reactive autoantigens and trigger autoimmune responses in hosts carrying high-risk human leukocyte antigen (HLA) genes [35,103]. For instance, numerous findings have suggested that gut commensal microbes may mimic retinal antigen(s), favoring the production of autoreactive T cells, triggering autoimmune uveitis [104]. In synovia and PBMCs from patients affected by rheumatoid arthritis, two autoantigens, N-acetylglucosamine-6-sulfatase and filamin A, targeted by T and B cells have been found [105]. Both antigens show high sequence homology with epitopes of some gut commensals, suggesting that immunological triggers at mucosal sites, such as the gut microbiota, may promote autoimmunity that affects joints, likely via the molecular mimicry mechanism [100]. A microbial peptide shared by several major classes of bacteria including *Escherichia coli*, which is one of the most common commensal bacteria of the human gut microbiota, can induce multiple sclerosis (MS)-like disease in humanized mice by cross-reacting with a T cell receptor that recognizes a peptide from myelin basic protein acting as candidate MS autoantigen [106]. Similarly, a peptide from *E. coli* has been demonstrated to induce autoimmune pancreatitis, likely by mimicking some self-antigens [107].

Increasing evidence suggests that a leaky gut is present in some, if not all, SLE patients, allowing pathogens and their products/metabolites to leak out from the gut lumen and penetrate the blood stream, reaching other organs and triggering inflammation and autoimmunity through the mechanism of molecular mimicry (Figure 1) [108]. The earliest anti-nuclear autoantibodies detected in SLE patients target the RNA-binding 60 kDa Ro protein and their production may be driven by Ro60 orthologs produced by commensal bacteria from different niches in genetic susceptible individuals through aberrant cross-reactive immune responses [109]. This hypothesis is supported by the observation that colonization of germ-free mice with *Bacteroides thetaiotaomicron* containing Ro60 ortholog caused T and B cell responses against human Ro60 and glomerular immune complex deposition [109]. The gut of SLE patients has an overall higher representation of *Ruminococcus gnavus* [83]. Anti-dsDNA autoantibodies cross-react with antigens from a *Ruminococcus gnavus* strain, contributing to the immune pathogenesis of LN, which suggests the possibility of developing a biomarker assay with diagnostic and prognostic value to assess the risk of LN [110]. In the sera from SLE patients, a significant positive correlation between higher titers of anti-*Enterococcus gallinarum* IgGs and the presence of autoantibodies, including anti-Ribosomal P (anti-Rib-P), anti-dsDNA, and anti-Smith (anti-Sm) autoantibodies, has been observed [111]. Moreover, *E. gallinarum* was detected in liver biopsies from lupus and autoimmune hepatitis patients, demonstrating that a gut pathobiont can translocate and promote autoimmunity in genetically predisposed hosts [112]. In a cohort of untreated SLE patients, numerous autoantigen-mimicking microbial peptides have been identified [81]. A peptide-mimicking human Fas antigen from *Akkermansia muciniphila* was found to bind to the IgGs produced by memory B cells from a subgroup of SLE patients, but not those from healthy controls [81]. *Bacteroides fragilis* is a Gram-negative obligate anaerobic bacterium of the normal human gut microbiota. *B. fragilis* ubiquitin (BfUb) shares 63% identity and more than 99% structural similarity with human ubiquitin (hUb) [113]. It has been reported that the sera from patients suffering from various ADs, including SLE and RA, contain higher levels of antibodies to BfUb compared to healthy volunteers, suggesting that molecular mimicry of hUb by BfUb could be a trigger for autoimmunity [113].

To date, no definitive data exist in the literature proving that the autoimmune response against endogenous Hsps in SLE patients may be caused by dysbiosis of the gut microbiota, accompanied by leaky gut, mediated by a molecular mimicry mechanism. However, this hypothesis is plausible because of the high similarity between human and bacterial Hsps. Moreover, numerous findings have demonstrated that the cross-reactivity between human and gut microbial Hsps is involved in the development of other autoimmune conditions. For example, IgG autoantibodies against human mitochondrial Hsp60 were significantly higher in the sera of patients with rheumatic autoimmune diseases, including SLE, than in healthy controls, and it was suggested that the antibodies were produced because GroEL, the *E. coli* Hsp60, shares immunogenic–antigenic epitopes with the mitochondrial chaperonin [114,115]. Microorganisms isolated from the jejunal mucosa of individuals affected by Kawasaki disease produce large amounts of Hsp60 and elicit the production of endogenous Hsp60 [116]. In turn, both bacterial and human Hsp60 molecules induce the activation of the immune system, triggering an inflammatory response against blood vessels typical of the disease [116]. It has been suggested that T cells specific to gut bacterial Hsps could cross-react against endogenous Hsps overexpressed in retinal ganglion cells and axons from glaucomatous mice and human glaucoma patients in response to elevated intraocular pressure, leading to progressive neurodegeneration in the eye [117].

A similar cross-reactivity mechanism between bacterial and human Hsps could cause the production of the autoantibodies against Hsp90 and Hsp70 found in SLE patients. The two chaperones occur both in bacteria and in humans with a high sequence similarity and are known to be immunogenic [118,119]. 

## 5. Conclusions and Future Perspectives

The SLE clinical manifestations can vary widely from individual to individual, ranging from milder symptoms to more severe and life-threatening ones. Because of this heterogenicity in phenotypes and clinical manifestations, which often mimic those of other conditions, and the lack of clear and robust diagnostic criteria, the diagnosis of SLE is still challenging, and the consequent diagnostic delay often prevents the timely choice of appropriate treatment, worsening both short- and long-term outcomes [120,121,122]. Therefore, identification of novel, strong, and unique biomarkers for early and accurate diagnosis could improve disease management and lead to personalized therapeutic interventions with tolerable side effects and curative results. To identify these biomarkers, knowledge of the factors involved in SLE pathogenesis is necessary.

Autoantibodies circulating in body fluids or forming immune complexes in peripheral tissues have been used as valuable diagnostic and prognostic biomarkers in SLE for predicting pathogenic pathways and for guiding therapeutic treatments [123,124]. Therefore, in recent years, several efforts have been made to improve the detection of autoantibodies. Synthetic peptides mimicking post-translationally modified autoantigens have been successfully used for the development of specific in vitro diagnostic/prognostic assays of autoimmune diseases, including SLE [125]. Moreover, the use of post-translationally modified peptides has allowed identification of autoantibodies associated with the most severe phenotypes [126].

Another way to make progress in this area is to research the immune mechanisms underlying the SLE pathogenesis. Here, we offer an overview of the involvement of two apparently independent and not interconnected factors in SLE etiopathogenesis, i.e., the CS and gut microbiota dysbiosis. Molecular mimicry could be the link between these two factors, whose pathogenicity in SLE is currently under scrutiny. Therefore, a comparison of primary and higher-order structures of components of the CS in human and gut microbes, which for instance may be facilitated by in silico analysis [127], could allow us to further elucidate the role of molecular mimicry in SLE. In this way, it may be possible to obtain new insights into disease pathogenesis and to develop novel and more efficacious therapeutic interventions that, for instance, could be based on the inhibition of the activity of the pathogenic Hsp(s) (negative chaperonotherapy).
